# Thermal and Hydraulic Performances of Carbon and Metallic Oxides-Based Nanomaterials

**DOI:** 10.3390/nano12091545

**Published:** 2022-05-03

**Authors:** Haitham Abdulmohsin Afan, Mohammed Suleman Aldlemy, Ali M. Ahmed, Ali H. Jawad, Maryam H. Naser, Raad Z. Homod, Zainab Haider Mussa, Adnan Hashim Abdulkadhim, Miklas Scholz, Zaher Mundher Yaseen

**Affiliations:** 1 Department of Civil Engineering, Al-Maarif University College, Ramadi 31001, Iraq; haitham.afan@uoa.edu.iq; 2 Department of Mechanical Engineering, College of Mechanical Engineering Technology, Benghazi 11199, Libya; maldlemy@ceb.edu.ly; 3 Center for Solar Energy Research and Studies (CSERS), Benghazi 11199, Libya; 4 Engineering Department, Al-Esraa University College, Baghdad 10011, Iraq; ali.majeed@esraa.edu.iq; 5 Faculty of Applied Sciences, Universiti Teknologi MARA, Shah Alam 40450, Selangor, Malaysia; ali288@uitm.edu.my; 6 Building and Construction Techniques Engineering Department, AL-Mustaqbal University College, Hillah 51001, Iraq; maryamhameed@mustaqbal-college.edu.iq; 7 Department of Oil and Gas Engineering, Basrah University for Oil and Gas, Al Basrah 61004, Iraq; raadahmood@yahoo.com; 8 College of Pharmacy, University of Al-Ameed, Karbala 56001, Iraq; za.alaady@alameed.edu.iq; 9 Department of Computer Engineering, Technical Engineering College, Al-Ayen University, Thi-Qar 64006, Iraq; adnan.hashin@alayen.edu.iq; 10 Division of Water Resources Engineering, Faculty of Engineering, Lund University, 221 00 Lund, Sweden; 11 Department of Civil Engineering Science, School of Civil Engineering and the Built Environment, University of Johannesburg, Kingsway Campus, Johannesburg 2092, South Africa; 12 Institute of Environmental Engineering, Wroclaw University of Environmental and Life Sciences, 50375 Wrocław, Poland; 13 Department of Town Planning, Engineering Networks and Systems, South Ural State University, 76, Lenin Prospekt, 454080 Chelyabinsk, Russia; 14 Department of Earth Sciences and Environment, Faculty of Science and Technology, Universiti Kebangsaan Malaysia, Bangi 43600, Selangor, Malaysia; 15 Adjunct Research Fellow, USQ’s Advanced Data Analytics Research Group, School of Mathematics Physics and Computing, University of Southern Queensland, Queensland, QLD 4350, Australia; 16 New Era and Development in Civil Engineering Research Group, Scientific Research Center, Al-Ayen University, Nasiriyah 64001, Iraq

**Keywords:** carbon nanostructures, metallic oxides, thermophysical properties, convective heat transfer, turbulent flow

## Abstract

For companies, notably in the realms of energy and power supply, the essential requirement for highly efficient thermal transport solutions has become a serious concern. Current research highlighted the use of metallic oxides and carbon-based nanofluids as heat transfer fluids. This work examined two carbon forms (PEG@GNPs & PEG@TGr) and two types of metallic oxides (Al_2_O_3_ & SiO_2_) in a square heated pipe in the mass fraction of 0.1 wt.%. Laboratory conditions were as follows: 6401 ≤ Re ≤ 11,907 and wall heat flux = 11,205 W/m^2^. The effective thermal–physical and heat transfer properties were assessed for fully developed turbulent fluid flow at 20–60 °C. The thermal and hydraulic performances of nanofluids were rated in terms of pumping power, performance index (PI), and performance evaluation criteria (PEC). The heat transfer coefficients of the nanofluids improved the most: PEG@GNPs = 44.4%, PEG@TGr = 41.2%, Al_2_O_3_ = 22.5%, and SiO_2_ = 24%. Meanwhile, the highest augmentation in the Nu of the nanofluids was as follows: PEG@GNPs = 35%, PEG@TGr = 30.1%, Al_2_O_3_ = 20.6%, and SiO_2_ = 21.9%. The pressure loss and friction factor increased the highest, by 20.8–23.7% and 3.57–3.85%, respectively. In the end, the general performance of nanofluids has shown that they would be a good alternative to the traditional working fluids in heat transfer requests.

## 1. Introduction

There was a lot of consideration in the production of working fluids with superior thermal characteristics to enhance heat transfer efficiency in heated pipes [1,2]. The latest research on nanofluids indicates that suspending extremely thermally conductive nanomaterials into the base fluid (e.g., water (DW) or Ethylene glycol (EG)) increases thermal conductivity, an increase in the base fluid’s convective heat transfer rate [3,4]. Reducing thermal boundary layer thickness generated by the existence of nanomaterials and their random movement within the base fluid might have a significant impact on such convective heat transfer coefficient augmentation [5,6]. An increase in the nanoparticle mass/volume concentration frequently improves the heat transfer rate of the base fluid. Adding more nanomaterials to the base fluid enhances the Brownian motion-driven variations in the fluid, which leads to a fast heat transfer from the wall to the nanofluid [7,8].

In the heat transfer and hydrodynamic applications, thermal conductivity shows a crucial role in evaluating nanofluid thermal efficiency, which has been calculated based on different factors, involving inlet temperature, nanomaterial types and mass percentage, the nanostructure of particles, base fluid properties, pH values, and types of surfactants/additives [9,10,11]. Additionally, dynamic viscosity influences the determination of heat and momentum transfer and the device’s pumping amount [12]. Meanwhile, less effort was dedicated to density, thermal expansion, and specific heat capacity [13]. The values of density and specific capacity of different nanofluids have been estimated through empirical correlations and equations based on the volume fraction of nanoparticles [14,15].

Numerous experimental and numerical investigation analyses evaluated forced convection heat transfer using different metal and metallic oxides such as Al, Cu, CuO, SiO_2_, Al_2_O_3_, TiO_2_, and MWCNTs during various flow regimes. Several mechanical or thermal equipment used constant wall heat flux (WHF) for heat transfer applications. Numerical and experimental efforts studied the effects of thermal and momentum diffusivity on the heat conductivity of various nano-powders under turbulent forced convective heat transfer [11]. The study examined various nanofluid samples (Al_2_O_3_-H_2_O, SiO_2_-H_2_O, and Cu-H_2_O) and various volume percentages (1–3 vol.%) at 30 °C. Improved thermal conductivity had no impact on heat transfer efficiency; but, the Prandtl number (Pr) of nanofluids significantly influenced the Nusselt number value at constant Re. Numerical and experimental analyses observed forced convective heat transfer by flowing graphene nanoplatelet nanofluids through a fully turbulent system inside a horizontally smooth heated pipe [16]. GNPs@H_2_O nanofluids increased from 7.96% to 25% in thermal efficiency. Furthermore, Nu_avg_ at 0.1 wt.% revealed enhancements of 75%, 79%, and 83% at 8231, 10,351, and 12,320 W/m^2^, respectively. Zubir and his group [17] produced reduced graphene oxide (R-GO) and its influence on a heat exchanger’s turbulent convective heat transfer performance. Furthermore, the study noticed substantial improvement in the Nusselt number up to 144% and 63% at the upstream and downstream of the test section, respectively. Laboratory work of PGGNPs with SSA-750 m^2^/g was carried out to assess nanofluid flow and heat transfer enhancement [18]. The test section was heated with two rates such as 23,870 and 18,565 W/m^2^. Meanwhile, the Re-number during the investigation ranged from 3900 to 11,700. From the results, the heat transfer coefficient improved significantly (around 119% & 84%) at the two heat rates. The performance index of all samples was larger than one, indicating that the synthesized PGGNP@DW nanofluids were effective for convective heat transfer. Yarmand et al. [19] reported the effect of pressure loss, thermophysical characteristics, and convective heat transfer on the stable-doped GNPs nanofluids. Their results showed positive improvements in both Nu-number and heat transfer coefficients by about 26.5% and 19.68%, respectively, at 0.1 wt.%. Lastly, Sadri et al. [20] prepared graphene nanoplatelets using green synthetization. They prepared three samples of C-GNPs nanofluids in 0.025, 0.075, and 0.1 wt.%. The results showed optimum improvements in the Nu-number (18.69%) and convective heat transfer coefficient (37.54%) at Re = 15,927 and 0.1 wt.%. It was clear that the performance index for all CGNP-DW nanofluids was larger than one. This demonstrated the advantage of employing environmentally friendly nanofluids in heat transfer systems. The overall thermal performance of using TiO_2_-DW nanofluids reached up to 1.519 as the best value, then reduced by increasing the nanofluid flow [21]. The thermal efficiency of SiO_2_-DW in a triangular tube with various turbulators was consistently greater than one. The index increased first with the increase in Re number and then decreased with it. This index reached its maximum at the Reynolds number Re = 6000 [22]. Additionally, in corrugated tubes, several working fluids (DW, GNP-SDBS@DW, Al_2_O_3_@DW, and SiO_2_@DW) and tube shapes (rectangular, triangular, trapezoidal, and curved ribs) were investigated [23]. The overall performance can be enhanced by up to 37% by combining the approaches (GNP-SDBS@DW nanofluids and curved pipe).

A closer look at the literature reveals several gaps and shortcomings of overall thermal performance using carbon and metal-oxide nanofluids within heated pipes. The main purpose was to compare the performance of functionalized carbon nanostructured nanofluids and commercial metallic oxides-based nanofluids. The prepared nanomaterials were characterized via different examinations to show successful chemical reactions. Meanwhile, the nanofluids’ thermo-physical properties of PEG@GNPs, PEG@TGr, Al_2_O_3_, and SiO_2_ were measured in the range of temperatures (20–60 °C). The heat transfer and nanofluids flow were evaluated based on several parameters such as the average Nu-number, relative pumping power, and different performance indicators under fully developed turbulent forced convective flow.

## 2. Materials and Methods

### 2.1. Functionalization and Preparation Process

**Figure 1:**
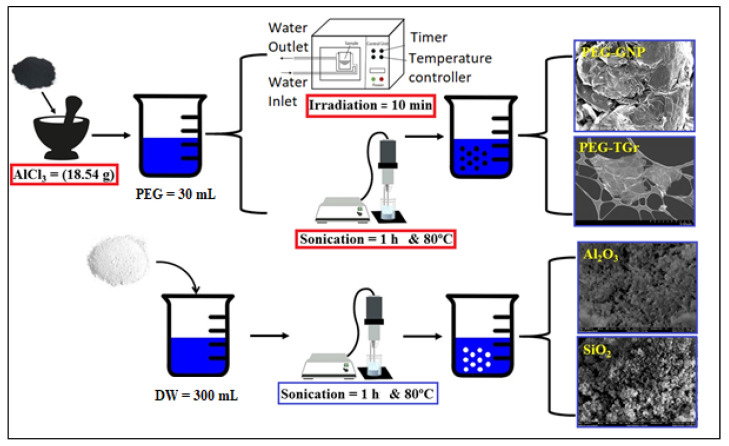
Schematic illustration for the different nanofluids preparation process.

Since the raw materials of GNPs and Gr are hydrophobic and cannot dissolve in polar solvents like H_2_O, a suitable way to make PEG@GNPs and PEG@TGr hydrophilic is to present the covalent functionalization via acid treatment. The process will dope the surface of GNPs and Gr with -OH- and -COOH. In a typical experiment [24], the chemical reactions were performed by dispersing GNPs (1 g) and Gr (1 g) in the acid medium of AlCl_3_ (18.54 g) and HCl (10 mL), followed by 1 h microwave radiation. Then, the solution was separated at 11,500 rpm and filtered through a polycarbonate filter (0.45 µm) before sequential washing with DMF, THF, diluted HCl, and enough DI-water to eliminate unreacted AlCl_3_ and PEG overnight at 60 °C. Furthermore, the dry aluminum oxide (Al_2_O_3_-NPs = 50 nm) and silicon dioxide (SiO_2_-NPs = 50 nm) were ultrasonicated for 1 h to avoid agglomeration/settlement. Nanomaterials were mixed with DW by an ultrasonic probe (Sonics Vibra-Cell, VC 750, Sonics & Materials Inc., Newtown, CT, USA) with an output power of (750 W) and a power supply of (20 kHz) frequency. The production process and nanofluid preparation method were shown in Figure 1 [25].

### 2.2. Experimental Methodologies

This study was carried out at an inlet temperature of 30 °C; the basic thermophysical properties such as dynamic viscosity and thermal conductivity should be determined initially. The tools of KD2 Pro and Anton Paar Rheometer were used to evaluate thermal conductivity and dynamic viscosity, respectively [26]. In the meantime, for the density readings, a density meter was used at an accuracy level of ±10^−4^ g/cm^3^. Lastly, a Differential Scanning Calorimeter (data accuracy = ±1.0%) was used to capture the specific heat of the samples. SEM-EDX analysis was conducted to study morphology and elemental structures of the prepared nanomaterials using VEGA3 tool (Tescan, Brno, Czechia).

**Figure 2:**
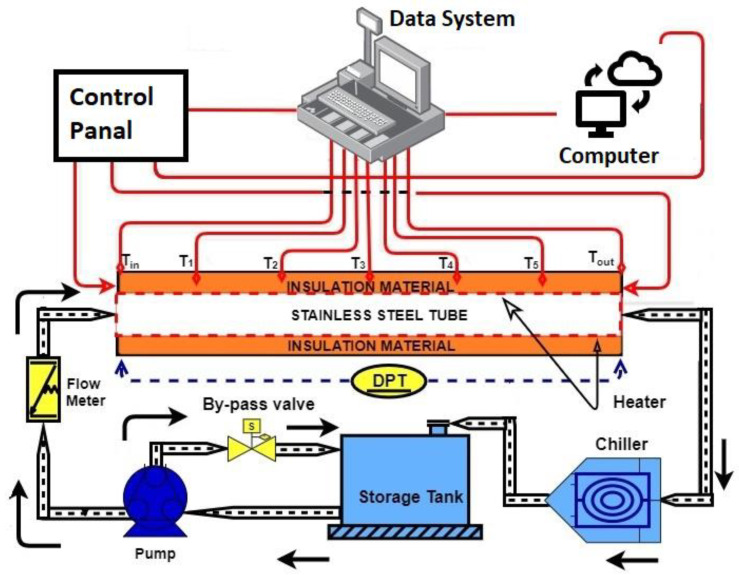
Diagram of the adopted experimental set-up.

Experimental model is schematically depicted in Figure 2. The flow loop parts include a magnetic flow meter, a storage tank, a pump, a test section, and a differential pressure transmitter. Each working fluid is driven from a 12 L capacity stainless steel by a magnetic drive pump at the flow rate range of 0–10 LPM. Uncertainties of the flow rate and pressure loss measures were ±0.5% and ±0.075%, respectively.

The test section is a square heated pipe (length = 1.4 m, inner width = 10 mm, outer width = 12.8 mm). It was heated by a 900 W flexible tape heater attached to a transformer and a power meter. Then, a high-temperature epoxy glue was used for installing 5 T-type thermocouples (uncertainty = ±0.1 °C) to measure the surface temperature.

Two RTD (PT-100) sensors (uncertainty = ±0.1 °C) were immersed into the pipe to measure the inlet and outlet temperatures. All temperature measurements were collected by Graphtec (LOGGER GL240). After using the formula ($Q=VI=\dot{m}C_{p}\left[T_{out}-T_{in}\right]$), the maximum heat loss was about 7.2%. This low heat loss rate was thought to have no significant impact on the entire process of heat transfer estimation.

### 2.3. Data Processing

In the current study, primary data were collected from an experimental setup and handled using very well-known procedures, as described in earlier studies [27]. The present laboratory analysis focused on evaluation of the heat transfer enhancement and hydrodynamic effectiveness under the condition of fully developed turbulent flow. The approximate heat flux, heat transfer coefficient, average Nusselt number, friction factor, Reynolds number, and Prandtl number; are presented as follows:
Heat flux (*q*″)$\frac{V\times I}{4D_{h}L}$(1)Heat transfer coefficient (*h*)$\frac{q^{\prime \prime }}{T_{w}-T_{b}}$(2)Nusselt number (*Nu*)$\frac{hD_{h}}{k}$(3)Friction factor (*f*)$\frac{\Delta P}{\left(\frac{L}{D}\right)\left(\frac{\rho v^{2}}{2}\right)}$(4)Reynolds Number (*Re*)$\frac{4\;\dot{m}}{\pi D_{h}\mu }$(5)Prandtl number (*Pr*)$\frac{\mu C_{p}}{k}$(6)

In this regard, $T_{w}=\frac{\sum T}{5}$. (*T_w_* = average wall surface temp.), $T_{b}=\frac{T_{o}-T_{i}}{2}$. $D_{h}=\frac{4A_{c}}{P}$, *A_c_* = cross-section area of square pipe, while *P* is the wetted perimeter.

The Gnielinski [28] relationship is justifiable, especially for the single-phase fluids flowing:

(7) $$Nu=\frac{\left(\frac{f}{8}\right)\left(Re-1000\right)Pr}{1+12.7{\left(\frac{f}{8}\right)}^{0.5}\left(Pr^{2/3}-1\right)}\left[1+{\left(\frac{d}{L}\right)}^{2/3}\right]{\left(\frac{Pr_{m}}{Pr_{w}}\right)}^{0.11}$$

where, $Pr_{m}$ = the bulk temperature-related Prandtl number and $Pr_{w}$ = wall temperature-related Prandtl number. The Gnielinski correlation remains valid in the range of 3000 < Re < 5 × 10^6^ and 0.5 < *Pr* < 2000.

The Colebrook equation [29] is applicable, based upon Re-number, in order to identify the friction factor of a fully developed turbulent flow using Equation (8).

(8) $$\frac{1}{\sqrt{f}}=-2.0\log\left(\frac{\varepsilon /D}{3.7}+\frac{2.51}{Re\sqrt{f}}\right)$$

Petukhov’s equation [30] of a fully developed turbulent flow is as shown in Equation (9):

(9) $$Nu=\frac{\left(\frac{f}{8}\right)RePr}{1.07+12.7{\left(\frac{f}{8}\right)}^{0.5}\left(Pr^{2/3}-1\right)}$$

Here, the formula is applicable for the requirements of 3000 < *Re* < 5 × 10^6^ and 0.5 < *Pr* < 2000.

The values of the Darcy friction factor were determined from the approximate pressure loss along the heated square pipe. The Blasius and Petukhov correlations were employed for the validation of the results obtained for the base fluid [31]:

Petukhov [30]:

(10) $$f={\left(0.79\ln\left(Re\right)-1.64\right)}^{-2}$$

Blasius [32]:

(11) $$f=\frac{0.316}{Re^{0.25}}$$

A performance index (*PI*) indicates an appropriate parameter to define various velocity and temperature ranges usable by various nanofluids [33]:

(12) PI=hnfhbf∆Pnf∆Pbf=RhR∆P

where (*R_h_*) is the ratio between nanofluids heat transfer and DW heat transfer, while (*R*_Δ*P*_) is the ratio between nanofluids pressure drop and DW pressure drop. An energy-saving indicator within the turbulent flow region calculated the pumping power using Equation (13).

(13) $$\frac{W_{nf}}{W_{bf}}={\left(\frac{\mu_{nf}}{\mu_{bf}}\right)}^{0.25}{\left(\frac{\rho_{bf}}{\rho_{nf}}\right)}^{2}$$

where $\left(W_{nf}\right)$ is the nanofluids’ pumping power and $\left(W_{bf}\right)$ is the DW pumping power.

The overall performance was evaluated (in terms of the thermal and hydraulic performances) using a performance evaluation criterion (PEC), which depicts the ratio of the heat performance to the nanofluids compared to DW. The formula of the PEC was expressed as [34]:

(14) PEC=NunfNubf(fnffbf)1/3

Table 1 presents and outlines the range of uncertainties [35].

## 3. Results and Discussion

### 3.1. Characterization of Nanofluids

**Figure 3:**
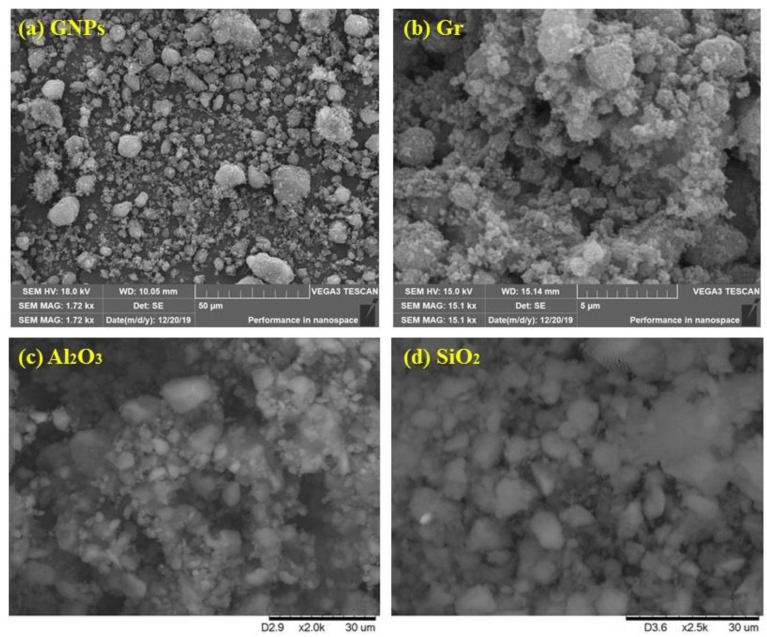
Visualization of different SEM nanoparticles; (**a**) Graphene nanoplatelets, (**b**) Graphene, (**c**) Alumina, (**d**) Silica.

SEM procedures are used to determine the content of particles based on the spectrum of the transmitted beam from the samples; they allow the size of irregularly sized particles or impurities to be determined. Moreover, SEM helps determine the distribution of nanoscale particles onto the surface of any sample. Figure 3a displays the SEM micrograph of the prepared PEG@GNPs; it is evident that the PEG@GNPs included several different-sized GNP-flakes, implying the samples’ high-purity level. Examination via the electron beam demonstrated that most flakes were transparent due to the limited number of their layers, though difficulties in determining precise flakes and defects diameter through SEM manifested themselves in sharper planar morphology of the GNP layers on the obtained SEM micrographs. Figure 3b displays the high-resolution SEM image of the PEG@TGr prior to any kind of pre-treatment. Also, consistency and intactness of the grains, curves, and wrinkling were observed on some of the transparent SEM images because of the strict production process. The existence of new functional groups in the PEG@TGr was revealed in observed functionalization-induced wrinkles in the images.

Figure 3c introduces the SEM image of the 0.1 wt.%-Al_2_O_3_@DW nanofluid; the image illustrates rod-like and rectangular-shaped alumina nanoparticles with a low tendency towards agglomeration of the excellence of the prepared suspension. Furthermore, the image demonstrated exceptional dispersal of the sample after 60 min of ultrasonication. Additionally, the nanoparticles demonstrated a homogeneous grain size (<50 nm), suggesting the prepared nanoparticles were spherical and showed a treatment-dependent size distribution. In the current study, it was observed from Figure 3c that the major bulk of the sample was Al_2_O_3_. This confirms the high purity of the sample and the suitability of the applied synthesizing methodology. Furthermore, Figure 3d shows the SEM image of 0.1 wt.%-SiO_2_@DW after 60 min ultrasonication nanofluid; the image shows the silica nanoparticles showing rod-to-round-like morphological, but with minor clusters and a better suspension. The size of nanoparticles was also found to be uniform, with <50 nm.

**Figure 4:**
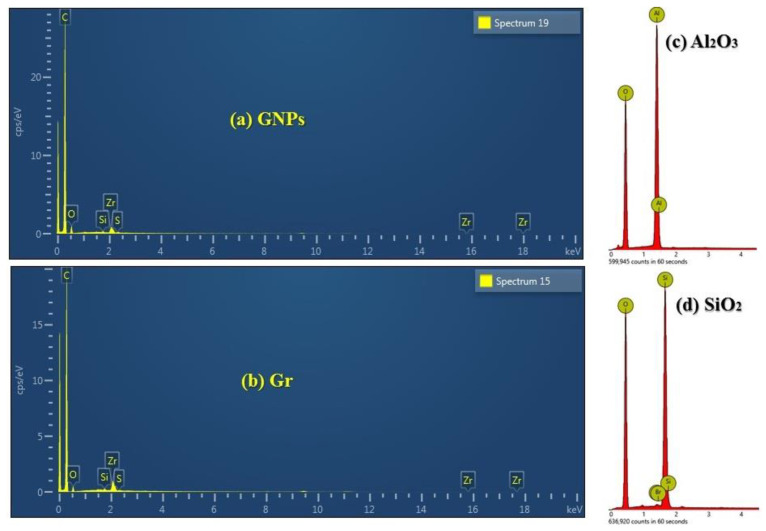
EDX images of different nanoparticles; (**a**) Graphene nanoplatelets, (**b**) Graphene, (**c**) Alumina, (**d**) Silica.

Figure 4 shows the EDX analysis for GNPs, Gr, Al_2_O_3,_ and SiO_2_ nanomaterials. As shown in Figure 4a,b, the carbon nanostructures show five elements (C, O, Si, S, and Zr). Figure 4c shows two elements only (Al and O). At the same time, Figure 4d presents three different elements (Si, O, and Br). The various elements refer to different synthesizing approaches used in this study.

### 3.2. Thermophysical Properties Measurements

**Figure 5:**
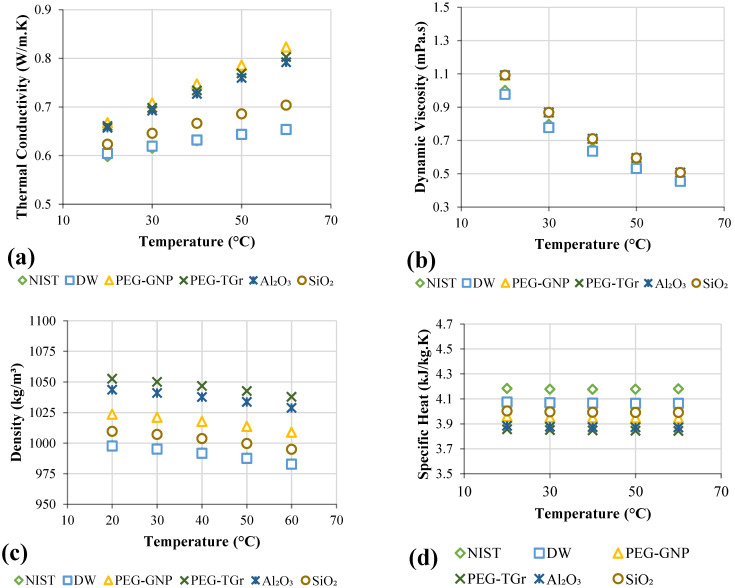
The thermophysical properties of base fluid and nanofluids; (**a**) Thermal conductivity, (**b**) Dynamic viscosity, (**c**) Density, (**d**) Specific heat capacity.

In comparison to distilled water, different nanofluids were described from the perspective of thermophysical properties as a function of mass fractions and temperature, as illustrated in Figure 5. The thermal conductivity of the working fluids plays a critical role in increasing heat removal efficiency from the heat exchangers to the environment. Current findings closely followed existing correlations offered by the National Institute of Science and Technology (NIST) [36], with a maximum standard error of 2%. As shown in Figure 5a, the nanofluids showed considerably higher thermal conductivity than DW; increases in temperature also rose thermal conductivity. The nano-coolants demonstrated a perfect, effective thermal conductivity increase rate at higher mass percentages. The temperature improved thermal conductivity significantly as a result of the increase in the nanoparticles’ Brownian motion upon DW. The increases in thermal conductivity were for PEG@GNP = 31.6%, PEG@TGr = 29.74%, SiO_2_ = 11.4%, and Al_2_O_3_ = 8.04% at 0.1 wt.% and 60 °C. Table 2 summarizes the thermal conductivity study by the previous investigators.

Figure 5b compared different nano-coolants and the base fluids in terms of their effective dynamic viscosity at the testing conditions of 0.1 wt.%, the temperature range of 20–60 °C, and a shear rate of 200 s^−1^. Figure 5b showed a minor increase in the nanofluids’ dynamic viscosity following that for DW, and the main reason for this increase is using low concentrations. It is assumed that fluid viscosity increases can result in pumping fluid penalty in the thermal applications; the nanofluids and DW also exhibited reduced dynamic viscosity due to the intermolecular forces degradation at increased temperatures [37]. The dynamic viscosity of all the samples showed a similar decreasing tendency, but the results evidenced increases in the base fluids’ dynamic viscosity. This validates the reliability of the proposed synthesis method for nanofluids in this study. Table 3 summarizes the dynamic viscosity study by previous researchers.

The density of the different working fluids was tested at a temperature range of 20 to 60 °C (see Figure 5c). The data showed a remarkable decrease in density with temperature and a slight increase in density with the nanofluid type. The nanoparticle’s density contributed to the improved density of the nano-coolants as it was higher than that of the base fluid. The observed improvement in the nanofluid density was as follows: PEG@GNP = 5.3%, PEG@TGr = 4.5%, Al_2_O_3_ = 2.6%, and SiO_2_ = 1.2% for 0.1 wt.% and 60 °C. However, the density reduced as follows: PEG@GNP = 1.7%, PEG@TGr = 1.8%, Al_2_O_3_ = 2.1%, and SiO_2_ = 2.7% after raising the temperature of the nanofluid from 20 to 60°C, thereby demonstrating the significant role of temperature.

Also, the specific heat capacities are measured in this study (see Figure 5d). The specific heat showed insignificant reductions with temperature increases, but the observed gradient concurred with the specific heat plots reported in the earlier studies [38]. Figure 5d evidenced the average specific heat decreases as follows: PEG@GNP = 5.4%, PEG@TGr = 4.8%, Al_2_O_3_ = 2.9%, and SiO_2_ = 1.8% compared to that of DW. This reduction was the lower specific heat of the solid nanoparticles relative to the base fluid.

### 3.3. Validation Test for Distilled Water

**Figure 6:**
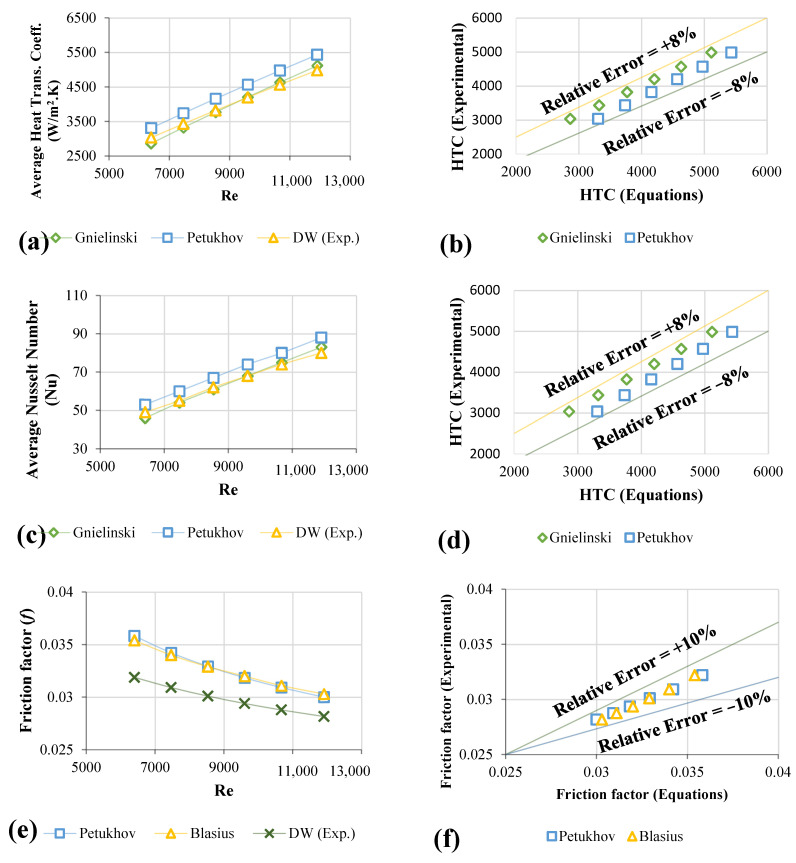

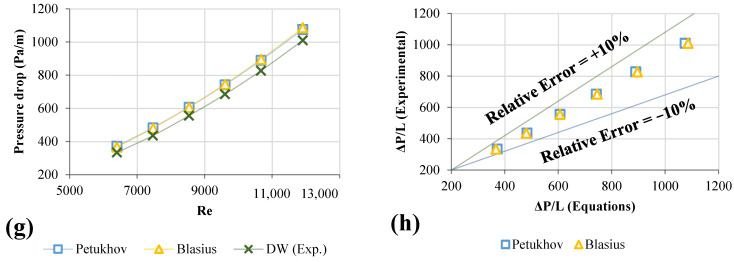
The verification assessment; (**a**) heat transfer coefficients measurement and prediction for 11,205 W/m^2^, (**b**) the magnitude of the relative error metric, (**c**) The value of the average Nusselt number at 11,205 W/m^2^, (**d**) the magnitude of the relative error metric, (**e**) Frictional head loss, (**f**) the magnitude of the relative error metric, (**g**) Pressure loss, (**h**) the magnitude of the relative error metric.

The Nu_avg_ and heat transfer coefficients (h) obtained with the data from Equations (7)–(9) are disclosed in Figure 6a–c. The data demonstrated outstanding agreement between the present findings and equations such as <8% with the Petukhov formula. The Gnielinski equation is better at low-range Re than the Petukhov equation at higher Re-values [43]. Figure 6b–d demonstrated the relative errors between the collected and equations data for average heat transfer coefficients and Nu_avg_.

Assessment of the experimental friction factor was calculated based on the measurement of the pressure loss in the entire applied heating pipe. The validation and verification procedures have been carried out using the Blasius and Petukhov equations for smooth pipes [44,45]. The validation of the experimental data for pressure loss and friction factor is shown in Figure 6e–g, while that of the data from the equations and the current study is shown in Figure 6f–h.

### 3.4. Convective Heat Transfer of Functionalized Nanofluids

The prepared samples in this study were made without adding surfactant due to their long-term stability [46]. The present study analyzed functionalized and commercial metallic oxide nanofluids to enhance convective heat transfer inside a square heat exchanger. Essentially, turbulent forced convective flow is typically conducted under heat transfer demands.

**Figure 7:**
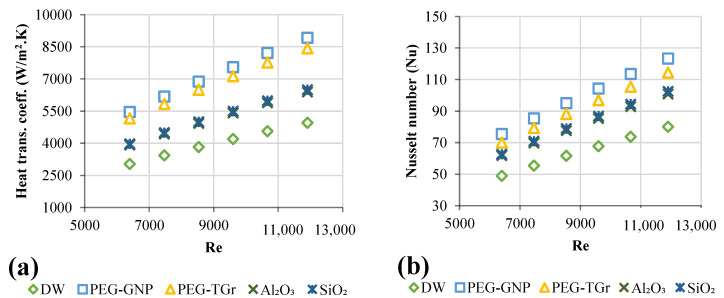
Heat transfer properties of different nanofluid types versus Reynolds numbers; (**a**) Heat transfer coefficients, (**b**) Average Nusselt number.

The convective heat transfer coefficients of the functionalized and metal oxides-based nanofluids are shown in Figure 7a versus multiple nanofluids and Re-numbers. Increased nanofluids convective heat transfer coefficient as the velocity of the working fluid increased. This improvement resulted from solid nanoparticles’ Brownian forces, thermal diffusion, and thermophoresis [47]. In the meantime, the increase in heat transfer might also result from the thin thermal boundary layer, which caused the higher velocities that caused thermal conductivity and decreased thermal resistance between the flowing nanofluid and the temperature of the internal wall surface of the heated pipe. Compared to DW, the maximum increase in heat transfer coefficients was as follows: PEG@GNPs = 44.4%, PEG@TGr = 41.2%, Al_2_O_3_ = 22.5%, and SiO_2_ = 24% at 0.1 wt.%. As per experimental data, the increase in heat transfer, as per test data, may be due either to the delay of the thermal boundary layers or due to increased thermal conductivity of the nanofluids.

Figure 7b introduced Nu_avg_ at 11,205 W/m^2^ and the Re-number function. The Nu_avg_ revealed an increase for each tested nanofluid. Observable higher Nu_avg_ of nanofluids reflected the decline in the circulation temperature after the working fluid had risen thermal conductivity; this subsequently reduced the temperature gradient between the wall of the tube and bulk fluid contained in the test-section. The maximum rise in Nu_avg_ was noted as follows: PEG@GNPs = 54%, PEG@TGr = 43%, SiO_2_ = 28%, and Al_2_O_3_ = 26% associated with DW.

### 3.5. Friction Factor of Nanofluids

**Figure 8:**
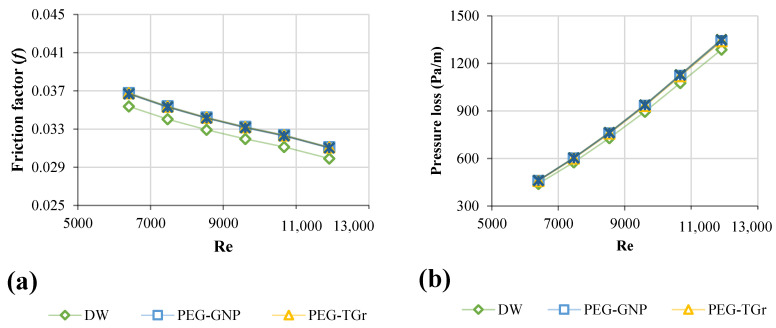
Hydrodynamic properties of different nanofluid types against Reynolds number; (**a**) Friction factor, (**b**) Pressure drop.

The nanofluids were evaluated for pressure loss and friction factor when flowing in a square heat exchanger at different Re-numbers. Figure 8a,b showed the measured pressure loss and friction factor for all samples versus the Re. The highest-pressure loss and friction factor increases were 20.8–23.7% and 3.57–3.85%, at a weight percentage of 0.1 wt. % and a velocity of 0.93 m/s, respectively.

Brownian motion significantly affects the momentum transfer between solid nanoparticles and base fluid molecules at a low range of Re-numbers. The friction factor of samples increases slightly due to the Brownian motion [48]. However, this is an inactive mechanism in the high Re range. Mainly, the velocity of the nanofluids can be considered the most significant factor for the development of friction factor at a high Re-number range. The considerable differences between the observed friction factors of functionalized carbon nanostructures, metallic oxides, and distilled water at multiple Re numbers are due to the minor improvement in the viscosities of distilled water and their nanofluids. The variations in the friction factor are based on the nanofluid-related viscous drag. Typically, the density of nanoparticles is a crucial factor in enhancing the nano-coolant friction factor. The combination of dynamic and kinematic viscosities dramatically affects the pressure drop of different nanofluids. The excessive pumping capacity increases with increased dynamic viscosity.

### 3.6. Performance Index and Performance Evaluation Criterion

**Figure 9:**
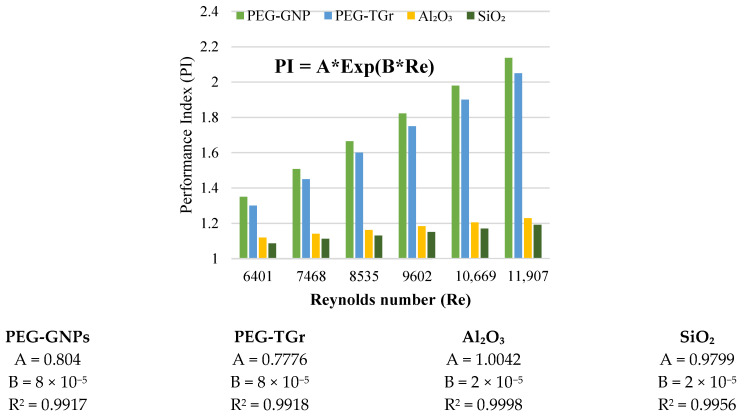
PI against different nanofluids and Reynolds number.

**Figure 10:**
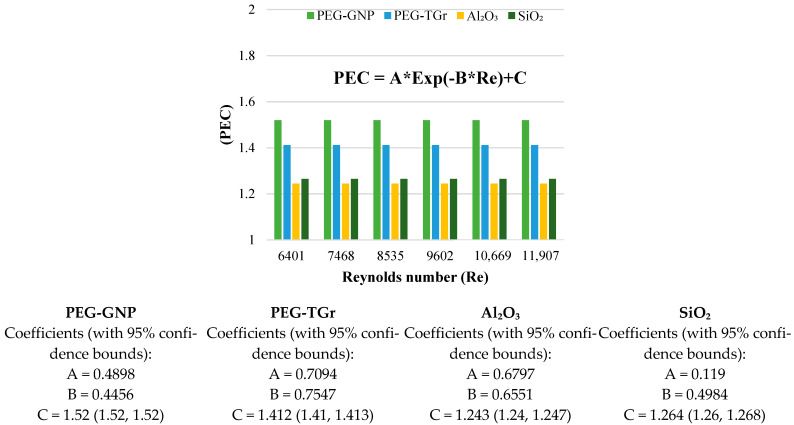
PEC of different nanofluids against Reynolds number.

Figure 9 and Figure 10 show changes in the performance characteristics of PI and PEC for different types of nanofluid vs. Re-numbers. The average PI, generally with PEC of the analyzed nanofluid, was noted to be >1 [34], indicating the effectiveness of the well-prepared nano-coolants for heated pipe flows. In addition, the carbon-based nanofluids presented higher augmentation of the metallic oxides due to a better rise in heat transport than the increased pressure loss. The PI of carbon and metallic oxides-based nanofluids improved with the Re-number; the maximum thermal efficiency of the nanofluids increased as follows: PEG@GNPs = 2.14, PEG@TGr = 2.05, Al_2_O_3_ = 1.23, and SiO_2_ = 1.19 at Re = 11,907, 0.1 wt.%, and 11,205 W/m^2^. This phenomenon was caused by the increased viscosity and thermal conductivity of nanofluids. The dynamic viscosity of a nanofluid can be increased to reduce the thickness of the boundary layer, resulting in an increase in heat transfer, while enhancing thermal conductivity enhances the thermal performance factor [18]. These results also confirm that the positive effects of heat transfer compensate for the negative impacts of pressure loss for carbon and metal-oxide nanofluids within a wide range of inlet temperatures, mass concentrations, and constant flow rates, stating that prepared nanofluids have excellent convective heat transfer capabilities.

Additionally, the findings of the PEC have shown a slight reduction in Re numbers. The maximum performance assessment of the nanofluids was as follows: PEG@GNPs = 1.52, PEG@TGr = 1.41, Al_2_O_3_ = 1.24, and SiO_2_ = 1.26.

### 3.7. Pumping Power of Different Nanofluids

**Figure 11:**
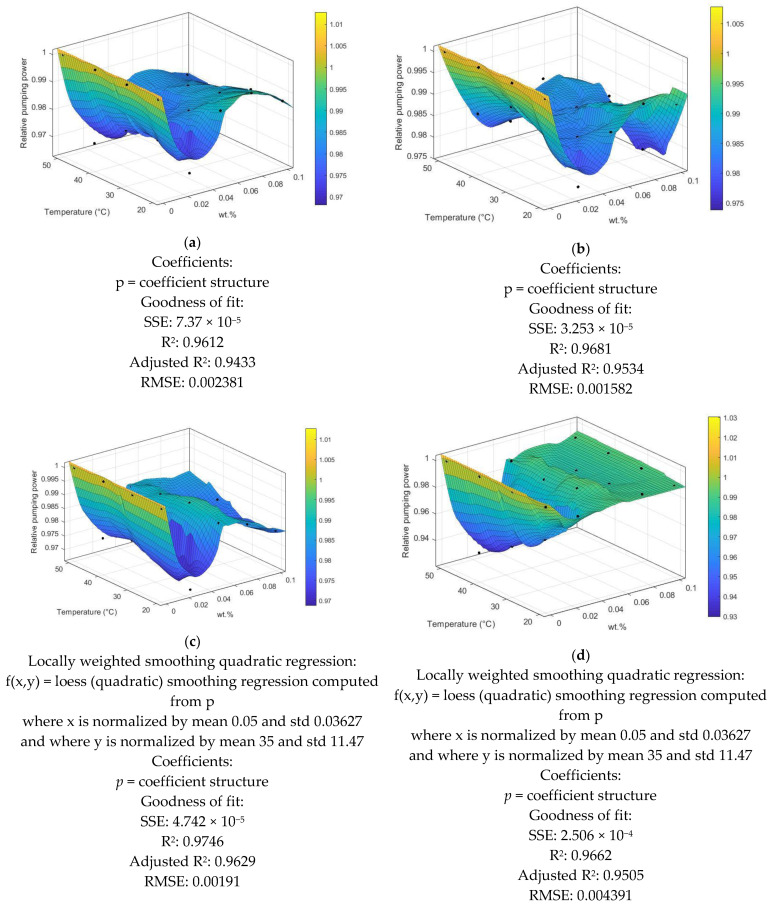
Relative pumping power of different nanofluids against different temperatures and various wt.%, (**a**) PEG@GNP, (**b**) PEG@TGr, (**c**) SiO_2_, and (**d**) Al_2_O_3_.

When choosing a heat exchanger, several criteria are the heat transfer rate, pumping power, cost, size and weight, type, and material. Friction effects in nanofluids cause pressure loss, and pressure loss calculations influence pumping power needs. Increased pumping power will result in more extraordinary capital expenses because larger pumps are more expensive and have higher operational costs due to the higher pumping power required. Pumping power measures a system’s financial ability to increase industrial and electrical energy. During the design of heat exchangers, it is essential to ensure low pumping power but effective heat transfer to ensure energy conservation. Figure 11 presents the pumping power for prepared nano-coolants at various Re with the working fluids. As the pumping power is dependent on the dynamic viscosity and density of both base fluid and the nanofluids (Equation (13)), the relative pumping power for all the tested samples is less than 1.

## 4. Conclusions

To improve the thermal performance of a square heat exchanger, four samples of nanofluids were covalently synthesized and produced. In order to start, all nano-coolants’ thermophysical properties were tracked versus the temperature to perform studies on the effects of heat and momentum transfer in fully developed turbulent forced convective flow. Different conditions were implemented, such as varying temperatures, different nanofluids, and different Re numbers.

From the results of this work, the following was concluded:

- The nanofluids exhibited the greatest thermal conductivity improvements as follows: PEG@GNPs = 31.6%, PEG@TGr = 29.74%, Al_2_O_3_ = 10.44%, and SiO_2_ = 9.32% at 60 °C and 0.1 wt.%.
- The highest improvement in heat transfer coefficients of the nanofluids was as follows: PEG@GNPs = 44.4%, PEG@TGr = 41.2%, Al_2_O_3_ = 22.5%, and SiO_2_ = 24 % at 0.1 wt.%. Meanwhile, the maximum enhancement in the Nu of the nanofluids was as follows: PEG@GNPs = 35%, PEG@TGr = 30.1%, Al_2_O_3_ = 20.6%, and SiO_2_ = 21.9% at 11,205 W/m^2^.
- The most significant pressure loss and friction factor increases were 20.8–23.7% and 3.57–3.85%, respectively. The effective dynamic viscosity significantly impacts the pressure drop for different nanofluids.
- The PI and PEC values of the tested samples were >1 and increased with the Reynolds number.
- Although the required pumping power was slightly increased, this was advantageous for the industrial application of these new working fluids.
- The nonlinear regression was developed for a relative pumping power of different nanofluids against temperature at different mass fractions.

## Figures and Tables

**Table 1:** Uncertainty ranges for heat transfer and fluid flow variables.

Variable	Uncertainty Equations	Uncertainty Values
Reynolds number, *Re*	$\frac{U_{Re}}{Re}=\sqrt{{\left(\frac{U_{\rho }}{\rho }\right)}^{2}+{\left(\frac{U_{\bar{V}}}{\bar{V}}\right)}^{2}+{\left(\frac{U_{\mu }}{\mu }\right)}^{2}}$	±1.73%
Constant Heat flux, *q*	$\frac{U_{q}}{q}=\sqrt{{\left(\frac{U_{V}}{V}\right)}^{2}+{\left(\frac{U_{I}}{I}\right)}^{2}}$	±1.51%
Heat transfer coefficient, *h*	$\frac{U_{h}}{h}=\sqrt{{\left(\frac{U_{q}}{q}\right)}^{2}+{\left(\frac{U_{\left(T_{w}-T_{b}\right)}}{\left(T_{w}-T_{b}\right)}\right)}^{2}}$	±1.52%
Nusselt number, *Nu*	$\frac{U_{Nu}}{Nu}=\sqrt{{\left(\frac{U_{h}}{h}\right)}^{2}+{\left(\frac{U_{k}}{k}\right)}^{2}}$	±5.23%
Friction factor, *f*	$\frac{U_{f}}{f}=\sqrt{{\left(\frac{U_{∆p}}{∆p}\right)}^{2}+{\left(\frac{U_{\rho }}{\rho }\right)}^{2}+{\left(\frac{U_{\bar{V}}}{\bar{V}}\right)}^{2}}$	±1.60%

**Table 2:** Summary of thermal conductivity in previous experimental studies.

Study	Nanofluid	Mass/Volume %	Base Fluid	Temp. Range	Tool	Remarks
[39]	Graphene (Gr)	0.005 and 0.01	Ionic Liquid	From 20 °C to 145 °C	Hot Disc-thermal constant analyzer	Thermal conductivity enhanced by 9.4% at 0.01 wt.%
[40]	Al_2_O_3_	0.2–1	DW+EG	From 10 °C to 50 °C	KD2pro	Thermal conductivity was enhanced by 8.3% at 1 vol.%
[41]	Graphene (Gr)	0.02–0.2	DW+EG	From 25–65 °C	KD2pro	Thermal conductivity enhanced by 64% at 0.2 wt.%
[42]	Graphene (Gr)	0.5–0.45	DW+EG	30 °C	KD2pro	Thermal conductivity enhanced by 18% at 0.45 vol.%
Current study	PEG@GNP, PEG@TGr, Al_2_O_3,_ and SiO_2_	0.1	DW	From 20 °C to 60 °C	KD2pro	PEG@GNP = 31.6%, PEG@TGr = 29.74%, SiO_2_ = 11.4%, & Al_2_O_3_ = 8.04% at 60 °C

**Table 3:** Summary of viscosity in previous experimental studies.

Study	Nanofluid	Mass/Volume%	Base Fluid	Temp. Range	Tool	Remarks
[39]	Graphene (Gr)	0.005 and 0.01	Ionic Liquid	From 25 °C to 150 °C	Viscometer	Viscosity enhanced by 29.1% and 13.4% raised for 0.005 and 0.01 wt.%
[40]	Al_2_O_3_	0.2–1	DW+EG	From 10 °C to 50 °C	Brookfield Viscometer	Viscosity and temperature were in opposite correlation
[41]	Graphene (Gr)	0.02–0.2	DW+EG	From 25–65 °C	Brookfield Viscometer	Viscosity decreases as temperature rises and increases as nanoparticle concentration rises.
Current study	PEG@GNP, PEG@TGr, Al_2_O_3,_ and SiO_2_	0.1	DW	From 20 °C to 60 °C	Anton Paar Rheometer	A minor increase in the nanofluids’ dynamic viscosity relative to DW.

## Data Availability

All the data are presented in the manuscript.

## Nomenclature

*A_c_*	Square Cross-section area of pipe, m^2^
*Al*_2_ *O*_3_	Alumina
*AlCl*_3_	Aluminum chloride
*CNTs*	Carbon nanotubes
*C_p_*	Specific heat capacity, kJ/kg K
*Cu*	Copper nanomaterials
*CuO*	Copper oxide
*D_h_*	Hydraulic Diameter of pipe, m
*DMF*	Dimethylformamide
*DPT*	Differential Pressure Transmitter
*DSC*	Differential Scanning Calorimeter
*DW*	Distilled water
*f*	Friction factor
*GNP*	Graphene Nanoplatelets
*GO*	Graphite oxide
*h*	Heat transfer coefficient, W/m^2^-K
*HCl*	Hydrochloric acid
*I*	Electric current, A
*k*	Thermal conductivity, W/m-K
*L*	Tube total-length, m
$\dot{m}$	Mass flow rate, kg/s
MWCNT	Multi-Walled Carbon Nanotube
*NIST*	National Institute of Standards and Technology
*Nu*	Average Nusselt number
*P*	Power supply, W
*P*	Perimeter of square pipe, m
*PEC*	Performance Evaluation Criterion
*PEG*	Pentaethylene Glycol
*PG*	Propylene Glycol
*Pr*	Prandtl number
*q*″	Constant Heat flux, W/m^2^
*Q*	Heat transfer amount, W
*RGO*	Reduced Graphene Oxide
*Re*	Reynolds number
*RTD*	Resistance Temperature Detector
*SiO*_2_	Silica nanomaterials
*T*	Temperature, °C
*T-Gr*	Thermally Treated Graphene
*THF*	Tetrahydrofuran
*TiO*_2_	Titanium dioxide
*U*	Velocity vector, m/s
*V*	Voltage, V
*v*	Working fluid velocity, m/s
*W*	Pumping power, W
**Greek symbols**	
*ρ*	Density, kg/m^3^
*μ*	Dynamic Viscosity, Pa·s
*ε*	Performance index
Δ*P*	Pressure loss
*φ*	Mass fraction, %
**Subscripts**	
*bf*	Base fluid
*nf*	Nanofluid
*np*	Particles in nanosize
*w*	Wall of pipe
*i*	Inlet
*o*	Output
*b*	Bulk fluid
